# Correlation between Prenatal Fetal Hemodynamic Parameters and Growth Indices in Pregnant Women with Preeclampsia and Neonatal Ultrasound Indicators of Organ Development

**DOI:** 10.12669/pjms.42.9.16320

**Published:** 2026-09

**Authors:** Shudan Yin, Jianhong Wang, Jinying Liu

**Affiliations:** 1 Shudan Yin, Department of Ultrasound, Xian People’s Hospital, Xi’an Fourth Hospital, Xi’an, Shaanxi Province 710004, P.R. China; 2 Jianhong Wang, Department of Ultrasound, Xian People’s Hospital, Xi’an Fourth Hospital, Xi’an, Shaanxi Province 710004, P.R. China; 3 Jinying Liu, Department of Ultrasound, Xian People’s Hospital, Xi’an Fourth Hospital, Xi’an, Shaanxi Province 710004, P.R. China

**Keywords:** Fetal Hemodynamics, Growth Parameters, Preeclampsia, Ultrasound, Organ Development

## Abstract

**Objectives:**

This retrospective study aimed to explore the associations between prenatal fetal hemodynamics and growth parameters in pregnant women with preeclampsia and neonatal ultrasound assessment indicators of organ development.

**Methodology:**

Clinical parameters of 296 pregnant women and their neonates admitted to Xi’an Fourth Hospital from December 2024 to December 2025 were collected. Those women diagnosed with preeclampsia were included in the study group (n=148). During the same period, age-matched healthy pregnant women and their neonates were included in the control group (n=148). Clinical data of prenatal and neonatal ultrasound examinations, fetal hemodynamic parameters, growth parameters, and neonatal organ development indicators were collected and analyzed.

**Results:**

Compared with the control group, the study group had significantly lower peak systolic velocity (PSV) and end diastolic velocity (EDV) of the umbilical artery, middle cerebral artery (MCA), renal artery (RA), biparietal diameter (BPD), head circumference (HC), abdominal circumference (AC), femur length (FL), bilateral renal volume, and liver thickness (P<0.05), whereas the myocardial performance index (MPI) was higher in the study group (P<0.05). Pearson correlation analysis showed that PSV, EDV, MCA, RA, BPD, HC, AC, and FL were positively correlated with bilateral renal volume and liver thickness, and negatively correlated with MPI (P<0.05).

**Conclusion:**

Preeclampsia was associated with insufficient fetal blood perfusion and growth retardation. Prenatal ultrasound indicators are closely related to neonatal developmental status and can be used to predict neonatal organ development.

## INTRODUCTION

Preeclampsia is a severe complication with high incidence in the second and third trimesters of pregnancy, with an occurrence rate of 5%–12%.^1^ The pathophysiology of preeclampsia is characterized by systemic vasospasm and endothelial dysfunction, which impair maternal circulation and result in inadequate placental perfusion, thereby reducing fetal oxygenation and nutrient delivery.^2^ Therefore, preeclampsia causes fetal growth restriction (FGR) and hemodynamic disorders and interferes with the maturation of fetal intrauterine organ development.^3^,^4^ The fetal period is a critical window for organ development. Therefore, adverse intrauterine environments, such as hypoxia and nutrient deficiency, can have long-term effects on the structure and function of key fetal organs, including the kidneys, liver, and heart, and increase the risk of long-term metabolic diseases.^5^–^7^ Therefore, clarifying the correlation between prenatal fetal hemodynamic parameters, growth parameters, and neonatal organ development indicators in pregnant women with preeclampsia is of great significance for early identification of fetal intrauterine developmental abnormalities, optimization of perinatal interventions, and improvement of the long-term prognosis of both mothers and infants.

Currently, clinical fetal monitoring in pregnant women with preeclampsia primarily relies on indicators such as fetal heart rate monitoring, routine measurements of growth diameters, and umbilical artery blood flow resistance, which do not fully reflect the status of fetal organ development.^8^,^9^ With the advancement of ultrasound technology, prenatal fetal hemodynamic detection (such as blood flow parameters of the middle cerebral artery and renal artery) and neonatal organ ultrasound assessment (such as renal volume, liver thickness, and myocardial performance index) have become important methods for accurate evaluation of developmental status.^10^,^11^ However, systematic research on the correlation between prenatal fetal hemodynamic parameters, growth parameters, and multi-organ ultrasound development indicators in the neonatal period for pregnant women with preeclampsia is still relatively scarce.

This study aimed to explore the correlation between prenatal fetal hemodynamic parameters and growth parameters in pregnant women with preeclampsia, and the association of these parameters with early postnatal ultrasound assessment of neonatal organ development.

## METHODOLOGY

Clinical data of 296 pregnant women and their neonates, delivered in Xi’an Fourth Hospital from December 2024 to December 2025, were retrospectively analyzed. Women with preeclampsia and their neonates were assigned to the study group. An age-matched control group was used, which enrolled 148 healthy pregnant women and their neonates with physical examination during the same period. The baseline demographic, obstetric, and laboratory characteristics of the study subjects are presented in Table-I. There was no statistically significant difference (P>0.05) in baseline data such as age, gestational age, pregnancy history, and newborn gender between the two groups of pregnant women, indicating comparability.

**Table-I:** Baseline characteristics of enrolled participants in the study and control group.

Group	No. of Cases	Maternal Age (years, *x̄*±*s*)	Gestational Age (weeks, *x̄*±*s*)	Maternal Type [n (%)]		Neonatal Gender [n (%)]		Apgar Score at 1 min (*x̄*±*s*)	Apgar Score at 5 min (*x̄*±*s*)
				Primipara	Multipara	Male	Female		
Study Group	148	29.63±4.58	37.25±1.86	92(62.16)	56(37.84)	76(51.35)	72(48.65)	8.96±0.75	9.59±0.42
Control Group	148	28.78±5.06	37.59±2.02	94(63.51)	54(36.49)	79(53.38)	69(46.62)	9.12±0.68	9.65±0.38
*t/χ*^2^ value		1.515	1.506	0.058		0.122		1.923	1.289
*P* value		0.131	0.133	0.810		0.727		0.056	0.199

### Ethical approval:

It was obtained from the Ethics Committee Ref. No. KJLL-Z-K-2026033, Approval date: February 10^th^, 2026)

### Inclusion Criteria:

- Diagnosis consistent with the established criteria for preeclampsia;12- Gestational age between 32 and 40 weeks, singleton pregnancy, live fetus, and complete clinical data.
- Maternal age between 20 and 40 years.
- Absence of preexisting conditions such as chronic hypertension, diabetes mellitus, or autoimmune diseases.
- Completion of prenatal ultrasound assessment of fetal hemodynamic and growth parameters.
- Neonatal survival after delivery with completion of renal, hepatic, and cardiac ultrasound examinations within 24-72 hours postpartum.
- Complete and traceable clinical records and ultrasound data without missing key variables.
- Inclusion Criteria for Control Group:
- Pregnant women who received prenatal care and delivered at our hospital during the same period.
- No history of pregnancy-related complications or comorbidities such as hypertension, diabetes mellitus, or placenta previa.
- Met the same eligibility criteria as the study group in all other respects.

### Exclusion Criteria:

- Presence of other pregnancy-related complications, including gestational diabetes mellitus, placenta previa, placental abruption, or intrauterine infection.
- Fetuses with chromosomal abnormalities, major structural malformations, or multiple gestations.
- Maternal history of long-term smoking, alcohol consumption, substance abuse, or exposure to toxic or harmful substances during pregnancy.
- Neonates who were preterm, had low birth weight, or experienced severe birth asphyxia.

### Fetal and neonatal examinations:

Prenatal and neonatal ultrasound data recorded in the electronic medical record system were retrospectively collected and analyzed.

### All groups underwent the following examinations:

### Prenatal fetal ultrasound examination:

A color Doppler ultrasound diagnostic instrument with a probe frequency of 3.5–5.0 MHz was used. The procedure was performed bysenior physicians with more than five years of experience in obstetric ultrasound according to the department’s standard clinical protocol.

Detection of fetal growth parameters was done by strictly following the Standards for Prenatal Ultrasound Examinations. The biparietal diameter (BPD) was measured as the vertical distance between the near and far field vertices of the fetal skull halo; the head circumference (HC) was measured as the maximum circumference of the fetal skull halo; the abdominal circumference (AC) was measured as the maximum abdominal circumference at the fetal umbilical level; the femur length (FL) was measured as the linear distance between the epiphyseal lines at both ends of the femoral shaft.

### Detection of fetal hemodynamic indicators:

The umbilical artery, middle cerebral artery, and renal artery were examined synchronously, with the angle between the probe and the blood flow direction ≤ 30°. The peak systolic velocity (PSV) and end diastolic velocity (EDV) were recorded.

### Neonatal Organ Ultrasound Examination:

A neonatal-specific ultrasound diagnostic instrument with a 5–10 MHz probe was used. The neonates were kept quiet; if necessary, nurses assisted in soothing them to avoid measurement interference from crying.

### Renal examination:

The neonates were placed in the supine or lateral decubitus position. Scanning was performed along the longitudinal and transverse sections of the kidneys. The bilateral renal length (linear distance between the upper and lower pole vertices), transverse diameter (maximum left-right diameter), and anteroposterior diameter (maximum anteroposterior diameter) were measured. The bilateral renal volume was calculated using the sphere volume formula (Volume = Length × Transverse Diameter × Anteroposterior Diameter × 0.523), and the average value of the two kidneys was taken as the final result.

### Hepatic examination:

The neonates were placed in the supine position. Scanning was performed through the right intercostal oblique section. The maximum thickness of the right hepatic lobe (vertical distance from the hepatic capsule to the inferior edge of the right hepatic lobe) was measured.

### Cardiac examination:

The neonates were placed in the supine position with their chests exposed. Scanning was performed using the parasternal long-axis view of the left ventricle and the four-chamber view. Pulsed Doppler ultrasound was used to measure the isovolumic contraction time (ICT), isovolumic relaxation time (IRT), and ejection time (ET). The myocardial performance index (MPI) was calculated as (MPI = (ICT + IRT) / ET), and the average of three measurements was taken.

### Observation indicators:

- Prenatal fetal hemodynamic parameters, including peak systolic velocity (PSV) and end-diastolic velocity (EDV) of the umbilical artery, middle cerebral artery, and renal artery.
- Prenatal fetal growth parameters, including biparietal diameter (BPD), head circumference (HC), abdominal circumference (AC), and femur length (FL).
- Neonatal organ development indicators, including bilateral renal volume, hepatic thickness, and myocardial performance index (MPI).
- Correlations between prenatal fetal hemodynamic parameters (PSV and EDV of the umbilical, middle cerebral, and renal arteries), fetal growth parameters (BPD, HC, AC, and FL), and neonatal organ development indicators (bilateral renal volume, hepatic thickness, and MPI) in the study group.

### Statistical analysis:

SPSS 22.0 software was used for data processing. Categorical variables were described as case numbers and analyzed by the chi-square (\chi^2) test. Continuous variables were tested for homogeneity of variance via the Bartlett test and for normality via the Kolmogorov-Smirnov test; after confirming homogeneity of variance and approximate normal distribution, the data were expressed as mean ± standard deviation (\bar{x}\pm s) and analyzed by the t-test. Pearson correlation analysis was performed to evaluate correlations. A value of P<0.05 was considered statistically significant.

## RESULTS

The baseline demographic and obstetric characteristics of enrolled participants are presented in Table-I. The age of the pregnant women in the study group ranged from 21 to 39 years, with a mean of 29.63±4.58 years. The gestational age ranged from 32 to 40 weeks, with a mean of 37.25±1.86 weeks. In the control group, the age of the pregnant women ranged from 22 to 38 years, with a mean of (28.78±5.06) years. The gestational age ranged from 33 to 41 weeks, with a mean of (37.59±2.02) weeks. There were no statistically significant differences in the baseline characteristics of the two groups, including maternal age, gestational age, obstetric history, and neonatal gender (P>0.05, Table-I).

Fetal hemodynamic parameters between the study group and the control group were compared (Table-II). The PSV and EDV of the umbilical artery, middle cerebral artery, and renal artery in the study group were all lower than those in the control group (P<0.05, Table-II).

**Table-II:** Comparison of Fetal hemodynamic parameters between the Study group and the Control group

Group	Case number	Umbilical Art		Middle Cerebral Artery		Renal Artery	
		PSV	EDV	PSV	EDV	PSV	EDV
Study group	148	47.85±7.23	17.32±4.38	56.26±8.15	20.53±4.72	35.57±3.96	12.85±2.42
Control group	148	51.37±6.59	19.75±3.96	60.18±7.43	23.46±4.28	38.24±5.47	13.68±3.15
*t value*		4.377	5.007	4.324	5.594	4.810	2.542
*P value*		<0.001	<0.001	<0.001	<0.001	<0.001	0.012

***Note:*** all indicators (cm/s) were shown as *x̄*±*s*

Fetal growth parameters between the two groups were compared (Table-III). The biparietal diameter (BPD), head circumference (HC), abdominal circumference (AC), and femur length (FL) in the study group were significantly lower compared to the control group (P<0.05; Table-III).

**Table-III:** Comparison of Fetal Growth Parameters between the Two Groups

Group	Case number	BPD	HC	AC	FL
Study group	148	83.37±4.52	311.25±16.48	298.53±15.62	65.28±3.75
Control group	148	86.54±3.89	316.82±14.26	305.78±13.94	66.85±4.12
*t* value		4.427	3.109	4.213	3.428
*P* value		<0.001	0.002	<0.001	0.001

***Note:*** all indicators (mm) were shown as *x̄*±*s*

Bilateral renal volume and liver thickness were lower in the study group than in the control group, whereas the myocardial performance index (MPI) was markedly higher (P<0.05; Table-IV).

**Table-IV:** Comparison of Neonatal Organ Development Indicators between the Two Groups

Group	Case number	Bilateral Renal Volume (cm3)	Liver Thickness (mm)	MPI
Study group	148	3.72±0.68	35.68±4.35	0.51±0.09
Control group	148	4.21±0.61	36.85±3.97	0.42±0.07
*t value*		6.525	2.417	9.603
*P value*		<0.001	0.016	<0.001

***Note:*** all indicators were shown as *x̄*±*s*

To further explore the associations between fetal hemodynamic parameters, the fetal growth parameters, and bilateral renal volume, liver thickness, and MPI, a correlation analysis was conducted. The results demonstrated that the PSV and the EDV of the umbilical artery, middle cerebral artery, and renal artery, as well as the BPD, HC, AC, and FL, were significantly positively correlated with bilateral renal volume and liver thickness, and significantly negatively correlated with the MPI (P<0.05, Table-V).

**Table-V:** Correlation Analysis.

Indicator		Bilateral Renal Volume	Liver Thickness	MPI
Umbilical Artery PSV	r/P Value	0.652/<0.001	0.562/<0.001	-0.408/<0.001
Umbilical Artery EDV	r/P Value	0.627/<0.001	0.539/<0.001	-0.385/<0.001
Middle Cerebral Artery PSV	r/P Value	0.704/<0.001	0.612/<0.001	-0.465/<0.001
Middle Cerebral Artery EDV	r/P Value	0.668/<0.001	0.587/<0.001	-0.432/<0.001
Renal Artery PSV	r/P Value	0.368/<0.001	0.315/<0.001	-0.287/<0.001
Renal Artery EDV	r/P Value	0.431/<0.001	0.376/<0.001	-0.335/<0.001
BPD	r/P Value	0.395/<0.001	0.342/<0.001	-0.306/<0.001
HC	r/P Value	0.467/<0.001	0.403/<0.001	-0.359/<0.001
AC	r/P Value	0.498/<0.001	0.445/<0.001	-0.389/<0.001
FL	r/P Value	0.543/<0.001	0.482/<0.001	-0.417/<0.001

## DISCUSSION

Preeclampsia and FGR are major causes of perinatal death and handicap in survivors. In preeclampsia, impaired remodeling of placental spiral arterioles increases vascular resistance and decreases blood flow velocity in the fetoplacental circulation.^13^ Even when prioritizing blood supply to the heart and brain, which is also called the “brain-sparing effect”, fetal circulation cannot compensate for reduced overall perfusion. As a result, the fetuses show decreased blood flow velocity in both core and peripheral blood vessels.^14^,^15^ This study investigated the changes of fetal hemodynamic parameters, the fetal growth parameters, and bilateral renal volume in preeclampsia. The results indicated that fetal PSV and EDV of the umbilical artery, middle cerebral artery, and renal artery in the preeclampsia group were significantly lower than those of the control group.

Previous research has confirmed that fetal umbilical artery resistance index and systolic-to-diastolic ratio in pregnant women with preeclampsia were significantly increased, while the blood flow velocity-related parameters were decreased, and the degree of abnormality was more obvious in severe cases.^16^ This is consistent with the conclusions of this study and further demonstrates that hemodynamic parameters can effectively reflect the state of fetal intrauterine hypoxia. However, that study only analyzed the umbilical artery without simultaneously evaluating the cerebral and renal arteries. On the other hand, the present study simultaneously assessed blood flow in multiple vessels, providing a more comprehensive reflection of the fetal circulatory status and compensating for the deficiencies of single-vessel assessment.

Fetal growth and development depend on a continuous and stable supply of nutrients. Therefore, insufficient placental perfusion leads to decreased protein synthesis and metabolic disorders.^17^ As the abdominal organs and skeletal system are particularly sensitive to nutritional deficiency, reductions in abdominal circumference and femur length are often more pronounced under conditions of impaired placental blood flow.^18^,^19^ A previous study by Yuan et al.^20^ showed that the estimated fetal weight of patients with preeclampsia complicated with FGR was significantly lower than that of patients with preeclampsia alone, and that the degree of abnormal glucose and lipid metabolism positively correlated with the severity of FGR. This finding further supports the mechanism by which preeclampsia inhibits fetal growth through dual pathways that affect maternal metabolism and placental perfusion, consistent with the abnormal growth parameters reported in this study.^20^ In addition, a study by Phillips JK et al.^21^ confirmed that the birth weight percentile of neonates born to women with early-onset preeclampsia was significantly lower than that of neonates born to patients with term preeclampsia. This study verified that the inhibitory effect of preeclampsia on fetal growth is correlated with the severity of the disease. This study also showed that preeclampsia was associated with reduced fetal growth parameters, such as BPD, HC, AC, and FL, suggesting that the adverse intrauterine environment may directly inhibit fetal somatic development.

Fetal renal development is characterized by blood flow dependence. Insufficient renal artery perfusion can directly affect nephron proliferation and renal parenchymal differentiation, resulting in a smaller neonatal renal volume.^22^,^23^ Previous research has demonstrated that during normal pregnancy, renal plasma flow and glomerular filtration rate increase substantially.^24^ In preeclampsia, maternal renal vasoconstriction reduces renal perfusion, accompanied by disruption of glomerular fenestrae and barrier function.^24^ However, another study revealed no significant difference in maternal renal perfusion between normal pregnancy and pre-eclampsia using 3D ultrasound.^25^ Neonatal ultrasound assessment in this study revealed that the bilateral renal volumes and the thickness of the right hepatic lobe were smaller in the preeclampsia group, whereas the MPI was higher. These results indicate that preeclampsia might affect neonatal organ development and functional status.

Intrauterine hypoxia induced by preeclampsia disrupts energy metabolism in cardiomyocytes, and the compensatory changes that develop during the fetal period persist into the neonatal period.^26^ A study by Wu G et al.^27^ showed that the global longitudinal strain and ejection fraction of the fetal left ventricle in pregnant women with gestational hypertension were decreased, and these cardiac function parameters were closely correlated with the risk of neonatal complications, which is consistent with the cardiac function abnormalities reflected by increased MPI in this study. The increased MPI in this study indicates an elevated cardiac functional load in neonates. Together, these results confirm the potential impact of preeclampsia on fetal cardiac development.

Fetal blood flow velocity directly reflects placental perfusion and organ blood supply, while growth parameters comprehensively reflect the overall fetal developmental level.^28^ Together, they form a dual-index system for evaluating the quality of the intrauterine developmental environment. For example, the EDV of the umbilical artery is an important indicator of placental vascular resistance. Lower EDV values reflect increased placental resistance and reduced perfusion, which may be associated with a higher risk of delayed development of neonatal renal and hepatic structures.^29^ AC is a candidate indicator for evaluating FGR.^30^ AC shows a strong correlation with neonatal liver and kidney volumes, further confirming the synchrony between fetal organ development and somatic growth.^31^ Correlation analysis in this study showed that prenatal fetal hemodynamic parameters (PSV, EDV) and growth parameters (BPD, HC, AC, FL) significantly positively correlated with neonatal bilateral renal volume and right hepatic lobe thickness, and significantly negatively correlated with MPI. These correlations suggested that prenatal ultrasound indicators have important clinical predictive value.

### Strengths:

This study provides novel evidence about the associations between prenatal fetal hemodynamics and growth parameters in pregnant women with preeclampsia, which might help identify specific targets for optimizing perinatal management in patients with preeclampsia. In cases where prenatal ultrasound reveals reduced fetal blood flow velocities and delayed growth parameters, timely adjustments to treatment, such as optimizing antihypertensive therapy and improving maternal hemodynamic management, may help improve placental perfusion. Furthermore, after delivery, neonates born to mothers with preeclampsia should undergo early organ ultrasound evaluation and functional monitoring to facilitate prompt detection of renal, hepatic, or cardiac abnormalities, thereby enabling timely intervention and potentially reducing short-term complications and long-term adverse outcomes.

### Limitations:

First, as a single-center retrospective analysis with a relatively small sample size, it is subject to potential selection bias, which may limit the generalizability of the findings. In addition, no sample size calculation or statistical power analysis was provided to support the reliability of the results. Second, ultrasound measurements inherently involve a degree of operator dependence, which may introduce measurement variability. Third, this study did not perform multivariable regression analyses to adjust for potential confounding factors, and no correction for multiple comparisons was applied. Certain potential confounding factors, such as undetected fetal chromosomal abnormalities and maternal nutritional status, were not comprehensively evaluated. Fourth, there is also a logical inconsistency in excluding cases of FGR while investigating growth retardation associated with preeclampsia. Fifth, this study did not investigate differences between early- and late-onset preeclampsia or between mild and severe cases, nor was long-term follow-up of neonatal organ function conducted.

## CONCLUSIONS

The results of this study suggest that preeclampsia was associated with insufficient blood flow perfusion in multiple organs of the fetus, overall growth and development retardation, and a continuous impact on the development and function of the liver, kidney, and heart of newborns. Prenatal fetal hemodynamic and growth parameters are significantly correlated with neonatal organ development indicators and associated with neonatal organ development indicators of poor neonatal organ development. Strengthening standardized prenatal ultrasound monitoring for pregnant women with preeclampsia is of great clinical significance for improving perinatal outcomes and reducing long-term health risks for newborns.

### Recommendations:

In future investigations, the associations between prenatal fetal hemodynamics and growth parameters in pregnant women with preeclampsia need to be validated using data from multicenter cohorts based on statistical power analysis. Further analysis should be performed upon addressing the impacts of geographic regions, different levels of obstetric care, and healthcare systems. In addition, additional laboratory indicators (e.g., blood lipid parameters, emerging molecular markers, angiogenic biomarkers, and so on), undetected fetal chromosomal abnormalities, and maternal nutritional status should be included for systematic analysis. Therefore, these variables can further improve the predictive performance and clinical applicability of clinical parameters. In terms of statistical analysis methods, univariate and multivariable regression analyses, nomograms, or machine learning techniques would provide better clinical reference by comprehensively integrating those biomarkers.

### Authors’ contributions:

**SY:** Literature search, study design and manuscript writing.

**JW and JL:** Data collection, data analysis and interpretation. Critical review.

**SY:** Manuscript revision and validation and is responsible for the integrity of the study.

All authors have read and approved the final manuscript.
